# Mask Use to Curtail Influenza in a Post–COVID-19 World: Modeling Study

**DOI:** 10.2196/31955

**Published:** 2022-05-27

**Authors:** Henri Froese, Angel G A Prempeh

**Affiliations:** 1 Goethe-University Frankfurt am Main Frankfurt Germany; 2 Saginaw Valley State University University Center, MI United States

**Keywords:** mask, protection, COVID-19, influenza, transmission, intervention, infectious disease, respiratory, simulation, model, prevalence, efficacy

## Abstract

**Background:**

Face mask mandates have been instrumental in the reduction of transmission of airborne COVID-19. Thus, the question arises whether comparatively mild measures should be kept in place after the pandemic to reduce other airborne diseases such as influenza.

**Objective:**

In this study, we aim to simulate the quantitative impact of face masks on the rate of influenza illnesses in the United States.

**Methods:**

Using the Centers for Disease Control and Prevention data from 2010 to 2019, we used a series of differential equations to simulate past influenza seasons, assuming that people wore face masks. This was achieved by introducing a variable to account for the efficacy and prevalence of masks and then analyzing its impact on influenza transmission rate in a susceptible-exposed-infected-recovered model fit to the actual past seasons. We then compared influenza rates in this hypothetical scenario with the actual rates over the seasons.

**Results:**

Our results show that several combinations of mask efficacy and prevalence can substantially reduce the burden of seasonal influenza. Across all the years modeled, a mask prevalence of 0.2 (20%) and assumed moderate inward and outward mask efficacy of 0.45 (45%) reduced influenza infections by >90%.

**Conclusions:**

A minority of individuals wearing masks substantially reduced the number of influenza infections across seasons. Considering the efficacy rates of masks and the relatively insignificant monetary cost, we highlight that it may be a viable alternative or complement to influenza vaccinations.

## Introduction

In March 2020, the World Health Organization officially declared COVID-19 a global pandemic, as it extended beyond borders and reached various parts of the world [1]. The spread of the virus has halted several activities and has placed uncertainty on future events. Scientists and researchers have recommended safety measures such as social distancing, wearing of masks, and quarantines to reduce infection rates or “flatten the curve” [2]. Fortunately, the mechanism of airborne infections has been well studied. In a social environment, oral fluid droplets filled with viral particles can travel from person to person [3]. Several studies indicate that the spread of such droplets can be reduced by facial coverings such as face masks [4]. As such, many governments have issued face mask mandates in public places in efforts to stop the spread of disease. In the advent of this new reality, recent analysis of respiratory specimens from 2018 to 2020 in Hong Kong indicate that rates of other respiratory pathogens such as respiratory syncytial virus and influenza are decreasing with increased mask-wearing [5]. This is not unique to Hong Kong; data from the United States, Australia, Chile, and South Africa also show significantly reduced rates of influenza following the widespread adoption of nonpharmaceutical interventions such as masks [6].

Noting the success achieved by this nonpharmaceutical measure, we ask if similar but less stringent measures should be kept in place after the COVID-19 pandemic to deal with influenza, which is another pertinent airborne disease.

To gain an in-depth and quantitative understanding of face masks’ impact on the reduction in influenza activity, we simulate how past influenza seasons 2010/2011 to 2018/2019 would have played out had people worn masks. The simulations were developed using deterministic compartmental models with the incorporation of variables to account for the impact of masks. Using publicly available influenza infection data for the past seasons from the Centers for Disease Control and Prevention (CDC), the influenzas transmission rates model for each season (2010/2011 to 2018/2019) was calibrated. We then simulated the seasons factoring in different scenarios of mask prevalence as well as inward-outward filtration efficacy of masks.

## Methods

### Susceptible-Exposed-Infected-Recovered Model and Parameters

Susceptible-exposed-infected-recovered (SEIR) models are a standard disease modeling technique in epidemiology. The population is compartmentalized into various groups: susceptible, exposed, infected, and recovered. Susceptible is the population susceptible to the disease. The exposed population are infected but have not been detected by testing. Infected is the population who have been confirmed to be infected and can transmit the disease. Recovered is the population who are recovered. To develop the SEIR model, the relationship between these groups is then mathematically characterized by differential equations. In our model, we used a basic SEIR model with a time-dependent transmission rate that is described by the following equations (Table 1):



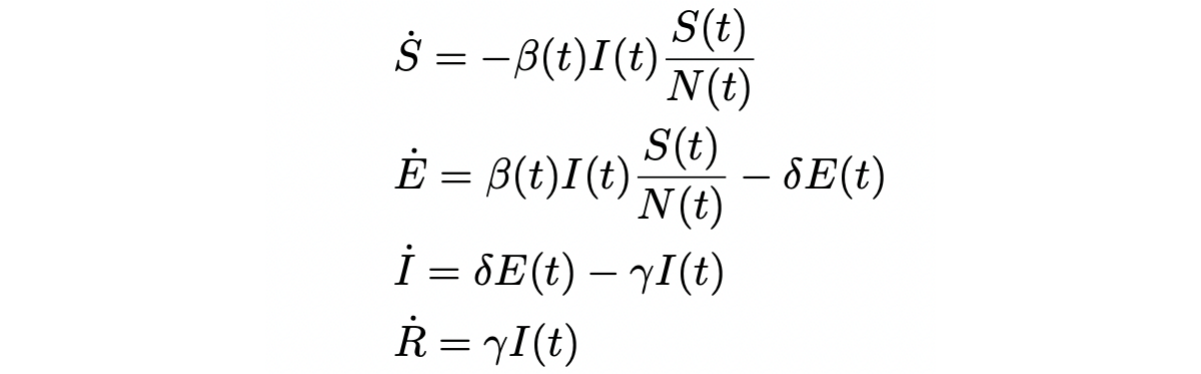



**Table 1:** Variables used in equations.

Variable	Parameter
S	Susceptible
E	Exposed
I	Infected
R	Recovered
β	Probability of disease transmission per contact times the number of contacts per unit time
δ	Rate of progression from exposed to infectious or inverse of the incubation period
γ	Rate of progression from infected to recovered or the inverse of the generation time
N	Total population (S + E + I + R)

Since the flu fatality rates are insignificant in relation to the total population [7], deaths from the flu and unrelated births and deaths were disregarded.

The transmission rate β(t) is described as the number of contacts an infected individual has per timestep, multiplied by the probability of disease transmission in a contact. Thus, as only 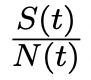 of the population can be infected, every infected individual infects β(t) 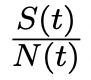 individuals per timestep.

In regard to influenza, all parameters of the SEIR model except the time-dependent transmission rate (β(t)) are publicly available via CDC data [8]. The CDC collects and compiles influenza activity year round in the United States. This is accomplished via the National Respiratory and Enteric Virus Surveillance System and the US World Health Organization Collaborating Laboratories System. This program consists of about 100 public health and 300 clinical laboratories throughout all 50 states, Puerto Rico, and the District of Colombia. All public health and clinical laboratories report the total number of tested specimen and the positive influenza tests. Since the influenza disease burden is based on testing and hospital reports, it is susceptible to underreporting. For example, there are cases where people with the flu may not report to the CDC or go see a health care provider. Therefore, to correct for this underreporting, the CDC uses a multiplier method with a routine population-based surveillance program to extrapolate a data set more representative of actual case rates [8].

We estimated β(t) by fitting the model to the scaled past infection data.

To account for mask use, a simplified version of the model used by Eikenberry et al [9] was adopted.

*m*_pre_*∈* [0*,* 1] is the mask prevalence, taken as the proportion of contacts in which an individual wears a mask. We assume that infection status does not affect mask-wearing behavior.

*m*_eff_*I ∈* [0*,* 1] is the efficacy of mask use by the infected individual (ie, the reduction of the chance of infection when only the infected individual wears a mask).

*m*_eff_*S ∈* [0*,* 1] is the efficacy of mask use by the susceptible individual (ie, the reduction of the chance of infection when only the susceptible individual wears a mask).

Consequently, we assumed that the reduction of the chance of infection when both individuals in a contact wear a mask is 1 *−* (1 *− m*_eff_*I*) · (1 *− m*_eff_*S*). For example, if the outward efficacy is 0*.*7 and the inward efficacy is 0*.*9, then the infection only happens in 3% of contacts where both individuals wear a mask. We combined the parameters to define the *mask impact m ∈* [0*,* 1], the proportion of contacts in which masks prevent an infection given the three parameters previously listed—that is, the sum of the proportions of contacts prevented if both individuals wear masks, only the infectious individual wears a mask, only the susceptible individual wears a mask, or no one wears a mask, leading to the following formula that sums these four cases up:



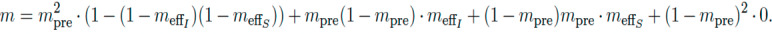



To incorporate *m* (the proportion of contacts prevented through mask use) into the model, note that without masks, every infected individual infects *β*(t) 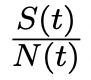 individuals per timestep—thus, with masks, this changes to (1 – *m*) ⋅ *β*(t) 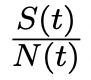, and we get the following model:



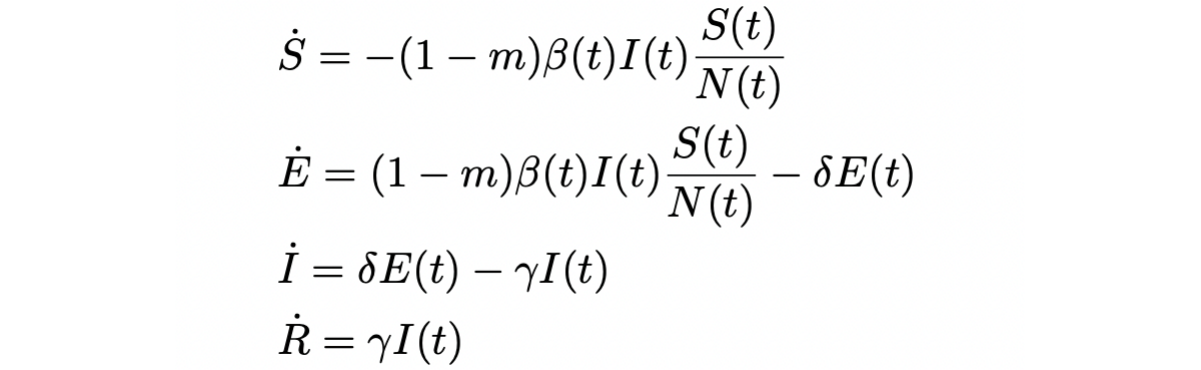



We will now look at the data used to fit *β*(*t*) for this model to past flu seasons (without masks; ie, with *m*=0).

### Infection Data

The CDC FluView application [10] provides weekly numbers of positive flu tests (we did not separate between different strains) in public health and clinical laboratories for the seasons 2010/2011 to 2018/2019. As mentioned previously, data from weekly numbers of infected individuals were extrapolated from weekly numbers of positive tests using the CDC’s estimated total number of infections per season.

For any season, let *P_i_* be the number of positive flu tests in week *i*. Let *T* be the total number of infections for the season. We assume that the number of positive tests is proportional to the actual number of infected *I_i_*, that is, *I_i_* = *λP_i_*, for all weeks *i*, for a fixed (per season) scaling factor *λ*>0. As infections persist on average, the sum of the infected per week over all weeks is (approximately) the total number of infections for the season:

*i I_i_*=*T*

Therefore, *i λP_i_* = *i I_i_* = *T* and the season’s scaling factor can be solved with:



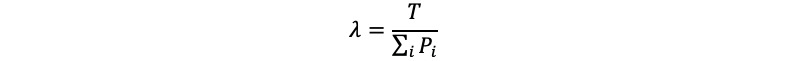



For each season, we calculated the scaling factor *λ* and used it to scale the CDC data.

### Beta Estimation From Infection Data

To estimate the time-dependent transmission rate, we fit a seasonal function of the form:



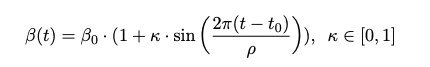



to the scaled data for each season, similar to the approaches by Towers and Feng [11] and Towers et al [12].

Timesteps *t* are in weeks. The incubation period and generation time are adapted from Mummert and Otunuga [13], yielding *γ*=1*.*0 (infections last 1 week) and 
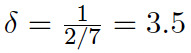
 (incubation period of 2 days).

**Figure 1:**
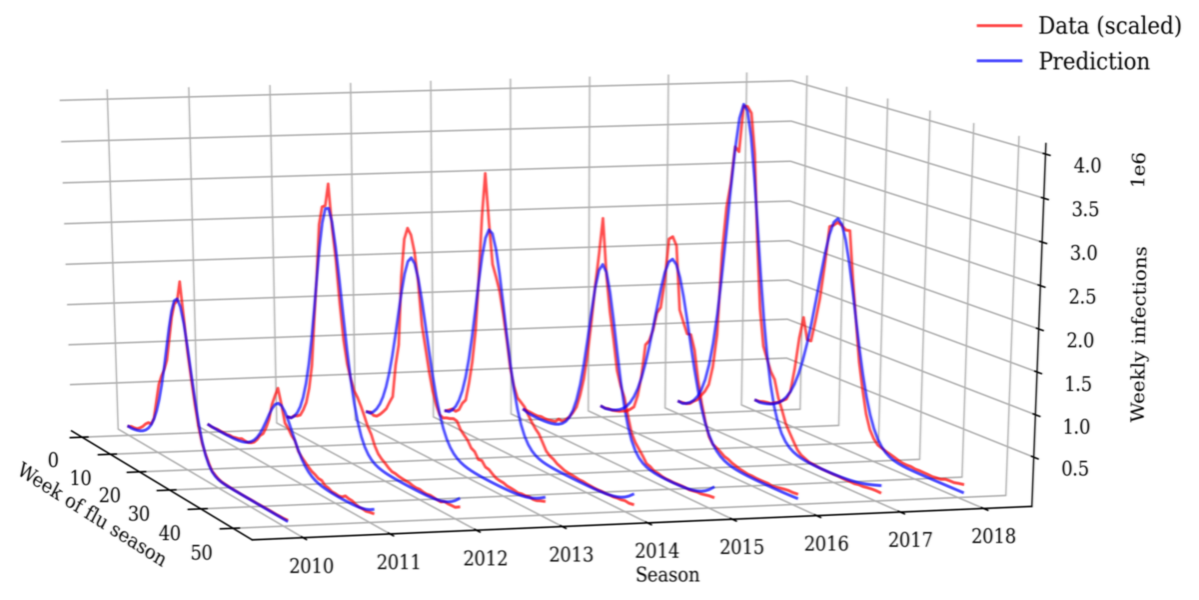
Results of the transmission rate fitting to data of past flu seasons. Actual infection data and prediction for influenza seasons 2010/2011 to 2018.

Least squares fitting using the LMFIT Python library [14] yielded good fits on all seasons (Figure 1).

## Results

**Figure 2:**
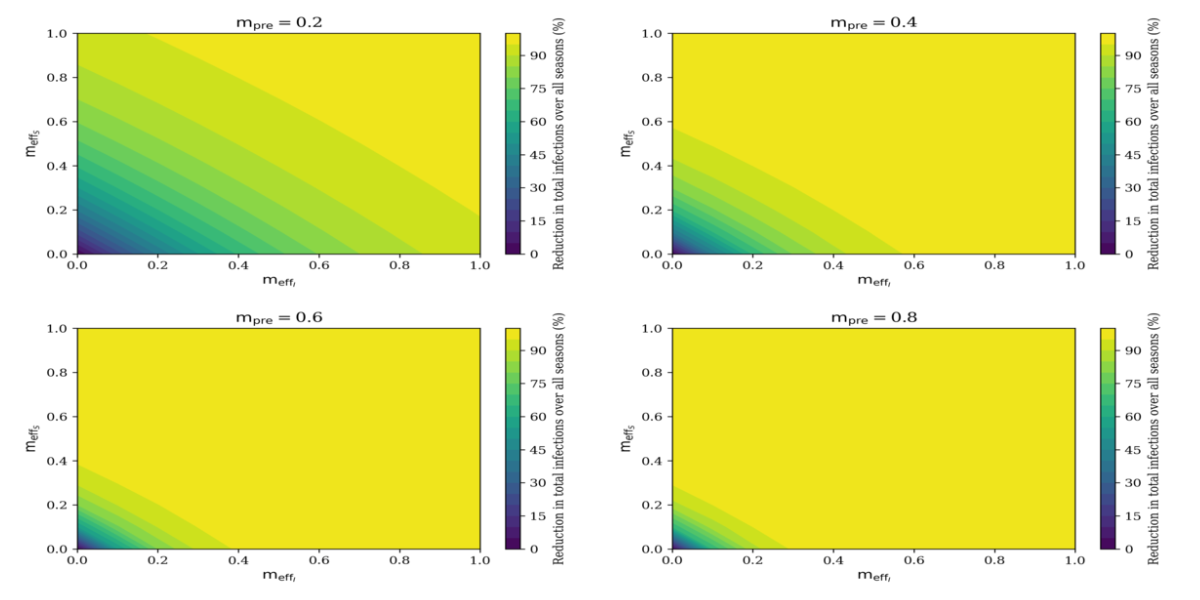
Reduction of total infections over all seasons pertaining to total infected mask wearing population and (meffi), and total susceptible mask wearing population (meffs) at mask prevalence levels (mpre) = 0.2 (20%), 0.4 (40%), 0.6 (60%) and 0.8 (80%).

We simulated the past influenza seasons with the estimated transmission rate *β*(*t*) and compare the outcome with and without masks. As evidenced by MacIntyre and Chughtai [15] and Brienen et al [16], mask efficacy is highly uncertain. Therefore, different combinations of mask prevalence and outward and inward efficacy were implemented (Figure 2).

From May to December 2020, mask use during the COVID-19 pandemic in the United States ranged from 50% to 70% [17]. Data from Pan et al [18] indicated that common fabrics such as a thin cotton bandana (two-ply) has a mask efficacy between 0.3 to 0.5 (30%-50%). We believe it is unlikely that mask prevalence will be as high after the COVID-19 pandemic without a mask mandate. Bearing this in mind, we look at two scenarios we deemed the most relevant: the *mask mandate* scenario with a mask prevalence of 0.5 (50%) and outward and inward efficacies of 0.35 (35%), and the *masks suggested* scenario with a prevalence of 0.2 (20%) and outward and inward efficacies of 0.45 (45%).

## Discussion

**Figure 3:**
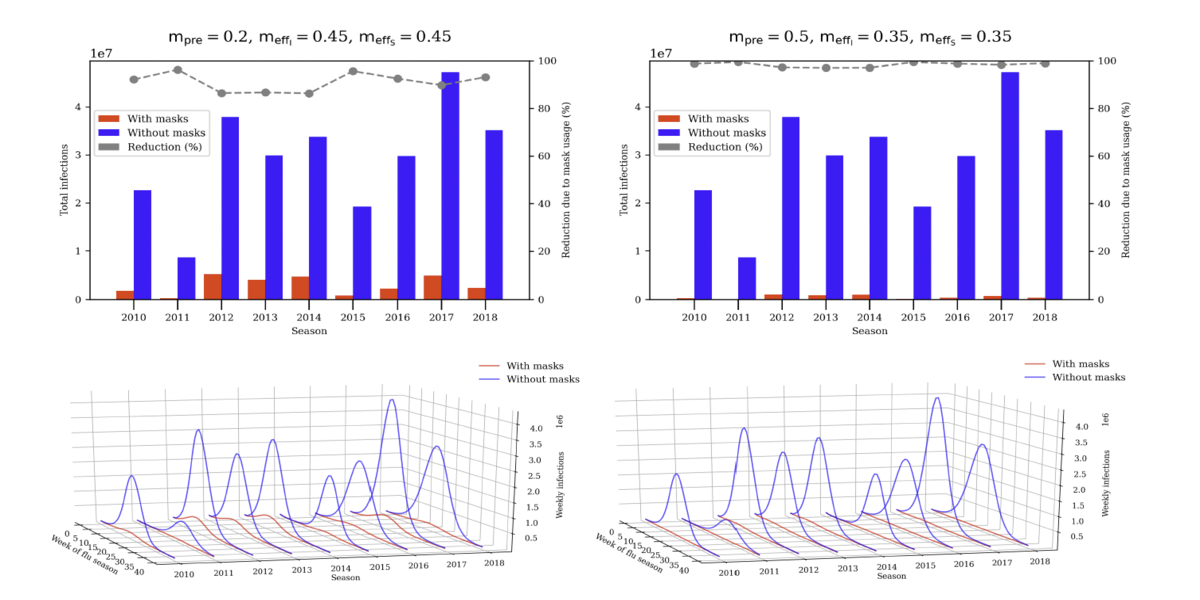
Simulated weekly infections for mask suggested scenarios (left) and mask mandate scenarios (right).

Our simulations showed that the “mask suggested scenario,” with relatively low mask prevalence of around 0.2 (20%) and assumed moderate inward and outward efficacy of 0.45 (45%), would have substantially reduced influenza infections by >90% over several past seasons. The “mask mandate scenario,” with 0.5 (50%) mask prevalence combined with an efficacy of 0.35 (35%), led to >95% reduction in influenza illnesses across seasons (Figure 3).

The findings show that when mask prevalence is high, for example, over 0.6 (60%), low mask efficacies (caused by masks worn too long, that are loose-fitting, etc) are sufficient to fully contain the flu. With that, it appears that a minority of disciplined mask wearers is sufficient to prevent most infection.

Currently, vaccinations are the prominent way to protect against influenza, having been available on a large scale since 1945 [13]. However, vaccination rates in the United States are not high enough to provide herd immunity [14]. In fact, flu vaccinations averted around 15% to 20% of influenza illnesses over the seasons from 2011/2012 to 2018/2019 [19]. Suggested data from this paper indicate that mask mandates in collaboration with vaccinations may be a more formidable tool against curbing influenza. Unlike masks, vaccines have to be newly manufactured each season with significant R&D investments. Nevertheless, vaccines only have to be administered once per year while face masks would need to be worn continuously. The continuous use of face masks in public spaces may be seen as more burdensome by the general population.

The economic burden of seasonal influenza in the United States is about US $6.3 to US $25.3 billion [20]. Assuming the economic cost scales linearly with the number of infections, a scenario in which at least 95% of infections are reduced (which includes both the *mask mandate* and *masks suggested* scenarios) saves US $6 to US $24 billion per season at negligible cost. Similar to public opinion regarding potential health hazards such as smoking and driving without seatbelts shifting over time and legislation being introduced, we can imagine the COVID-19 pandemic changing public (and expert) opinion toward everyday mask use. Although, large parts of the population might be tired of wearing masks after the COVID-19 pandemic. Public opinion shifts, but at least a minority of individuals may wear masks. Our simulations show that this would substantially reduce the burden of seasonal influenza at little monetary cost.

The limitations of our approach include no stratification by age or contact scenario, significant uncertainties in mask use and efficacy, and disregard of other nonpharmaceutical interventions.

## Abbreviations

CDC: Centers for Disease Control and Prevention
SEIR: susceptible-exposed-infected-recovered
